# Prognostic factors and long-term outcome of autologous haematopoietic stem cell transplantation following a uniform-modified BEAM-conditioning regimen for patients with refractory or relapsed Hodgkin lymphoma: a single-center experience

**DOI:** 10.1007/s12032-013-0611-y

**Published:** 2013-05-24

**Authors:** Anna Czyz, Anna Lojko-Dankowska, Dominik Dytfeld, Adam Nowicki, Lidia Gil, Magdalena Matuszak, Maria Kozlowska-Skrzypczak, Maciej Kazmierczak, Ewa Bembnista, Mieczysław Komarnicki

**Affiliations:** Department of Hematology, Poznan University of Medical Sciences, Szamarzewskiego 84, 61-569 Poznan, Poland

**Keywords:** Hodgkin lymphoma, Autologous haematopoietic stem cell transplantation, BEAM, Prognostic factors, Lymphocyte recovery, Positron emission tomography

## Abstract

Despite the well-defined role of autologous haematopoietic stem cell transplantation (autoHCT) in the treatment of patients with relapsed or refractory Hodgkin lymphoma (HL), relapse remains the main cause of transplant failure. We retrospectively evaluated long-term outcome and prognostic factors affecting survival of 132 patients with refractory (*n* = 89) or relapsed HL (*n* = 43) treated with autoHCT following modified BEAM. With a median follow-up of 68 months, the 10-year overall survival (OS) and progression-free survival (PFS) were 76 and 66 %, respectively. The 10-year cumulative incidence of second malignancies was 7 %. In multivariate analysis, age ≥45 years, more than one salvage regimens and disease status at transplant worse than CR were factors predictive for poor OS. In relapsed HL, age at transplant, response duration (<12 vs. ≥12 months) and the number of salvage regimens were independent predictors for PFS. In the refractory setting, disease status at autoHCT and the number of salvage regimens impacted PFS. The number of risk factors was inversely correlated with PFS in both relapsed and refractory HL (*p* = 0.003 and <0.001, respectively). The median PFS for patients with >1 risk factor in the relapsed and refractory setting was 5 and 11 months, respectively, in comparison with the median PFS not reached for patients with 0–1 risk factor in both settings. We conclude that high proportion of patients with relapsed/refractory HL can be cured with autoHCT. However, the presence of two or more risk factors helps to identify poor prognosis patients who may benefit from novel treatment strategies.

## Introduction

During the last decades, the development of efficient combination chemotherapy and more appropriate radiotherapy has improved overall long-term survival from Hodgkin lymphoma (HL) with preserving the balance between treatment high efficacy and acceptable toxicity. With modern up-front therapy, complete remission rate exceeds 80–85 %. However, about 15–20 % of patients with advanced HL do not achieve CR, and in addition, approximately 20–25 % of patients are expected to relapse at different time intervals from complete remission. High-dose therapy (HDT) followed by autologous hematopoietic stem cell transplantation (autoHCT) is considered the standard treatment recommended by available guidelines for patients with relapsed or refractory HL [1, 2]. This treatment provides long-term disease-free survival in over 50 % of patients [3–5]. Unfortunately, approximately 30 % of patients develop a recurrence after autoHCT [6, 7]. The prognosis in postransplant relapsed setting is poor [7, 8]. Therefore, the identification of patients with high risk of relapse after autoHCT is important, since new treatment strategies with novel agents are evaluated in ongoing studies. Clinical features that are considered important survival predictors include first response duration, the number of salvage therapy lines, chemosensitivity before autoHCT, age, presence of extranodal disease, B symptoms and anemia [5, 9–13]. However, the results of published studies on risk factors predicting survival after autoHCT revealed some discrepancies. More recently, the role of prefunctional imaging (FI) with 18F-fluoro-deoxy-d-glucose positron emission tomography (^18^FDG-PET) has been intensively investigated. There has been reported some evidence which proves that the negative ^18^FDG-PET status may be an independent determinant of favorable outcome after autoHCT [14–18].

To enhance the published experience, we conducted a retrospective review and present our single-center experience of patients who underwent autoHCT following modified BEAM preparative regimen for refractory or relapsed HL. We intended to report the long-term outcome and to define the prognostic factors that influenced outcome after autoHCT. Herein, we report the results of this analysis.

## Patients and methods

### Study population

We retrospectively reviewed the data of all patients with refractory or relapsed HL who were treated with modified BEAM regimen followed by autoHCT between January 2001 and December 2011 at our center. Refractory disease was defined as active disease (response worse than complete remission) after first-line chemotherapy or relapse within 3 months of its completion. Patients with relapsed disease were those who relapsed after at least 3 months of complete remission achieved with frontline therapy.

Patients records were reviewed to obtain patient characteristics and treatment details (clinical stage according to the Ann Arbor system, presence of B symptoms, the type of first-line chemotherapy, response to first-line chemotherapy, the duration of remission, the number and type of salvage chemotherapy lines, radiotherapy before autotransplant, disease status at transplant, absolute lymphocyte count (ALC) before starting HDT, ALC at 15 ± 1 day following autologous stem cell infusion). Complete response (CR), partial response (PR), stable disease (SD) and disease progression were defined using standard criteria [19]. Pretransplant evaluation and re-evaluation after transplant included physical examination, computed tomography (CT), blood count, chemistry evaluation and bone marrow biopsy in patients with bone marrow involvement at diagnosis or at relapse/progression. Pretransplant ^18^FDG-PET has been performed routinely since May 2008. Patients provided informed consent for the treatment.

### Transplant procedures

Patients underwent hematopoietic cell collection either by bone marrow harvest or by leukapheresis following stem cell mobilization. Stem cell mobilization was performed using salvage chemotherapy or cyclophosphamide (4 g/m^2^) ± etoposide (600 mg/m^2^) with G-CSF stimulation. The stem cells were cryopreserved without further manipulation. The high-dose modified BEAM regimen consisted of carmustine (total dose 300 mg/m^2^), etoposide (total dose 800 mg/m^2^), cytarabine (total dose 6,000 mg/m^2^), melphalan (total dose 140 mg/m^2^) and dexamethasone (total dose 168 mg/m^2^).

### Statistical analysis

Survival curves were estimated according to the method of Kaplan and Meier. Overall survival (OS) was measured from the time of transplantation until death from any cause, and progression-free survival (PFS) was measured from the time of transplantation until documented progression or relapse or death from any reason. Non-relapse mortality (NRM) was defined as death from any cause other than lymphoma relapse/progression. The probabilities of NRM, relapse and second malignancy were calculated with the cumulative incidence estimator. The cumulative incidence of NRM and relapse was calculated with either relapse- or non-relapse-related mortality treated as competing risk. The cumulative incidence of second malignancy was calculated in the survivors’ group, with death from any reason other than second neoplasm treated as a competing risk.

The two-tailed log-rank test was utilized to compare the curves. *p* values <0.05 were considered significant. Potential prognostic factors, age, clinical stage, presence of B symptoms, a duration of remission, a total number of salvage chemotherapy lines before autoHCT, radiotherapy prior to transplant, ALC at transplant, disease status at transplant and ALC at 15 ± 1 day after stem cell infusion were evaluated for OS and PFS in univariate analysis. Cox proportional hazards model was used for multivariable analysis.

SPSS version 14.0 (SPSS, Chicago, IL) was used for all statistical analyses except of cumulative incidence curves analyses, which were calculated using the statistical package NCSS version 2007 (NCSS, Kaysville, UT).

## Results

### Patients characteristics, prior treatment and transplantation procedures details

From January 2001 to December 2011, the 132 patients (71 men and 61 women) with refractory (*n* = 89) or relapsed (*n* = 43) HL underwent autoHCT following modified BEAM-conditioning regimen. Patient baseline characteristics and treatment details are presented in Table 1.

**Table 1:** Patient characteristics and treatment details

Characteristics	Number (%)
Total number of pts	132 (100)
Age (years)	
Median 42, range 15–64	
<45 years	111 (84)
≥45 years	21 (16)
Gender	
Male	71 (54)
Female	61 (46)
Clinical stage	
II	28 (21)
III	33 (25)
IV	67 (51)
Unknown	4 (3)
Constitutional symptoms	
Absent	32 (24)
Present	94 (71)
Unknown	6 (5)
Induction chemotherapy	
ABVD	108 (82)
BEACOPP or escalated BEACOPP	14 (11)
MOPP	6 (4)
Other regimens	4 (3)
Duration of remission in group of patients with relapsed disease (*n* = 43)	
<=12 months	20 (46.5)
>12 months	23 (53.5)
Second-line chemotherapy	
ESHAP or DHAP	120 (91)
Escalated BEACOPP	10 (7.5)
Other regimens	2 (1.5)
Number of pretransplant salvage chemotherapy lines	
1	86 (65)
>1	46 (35)
Radiotherapy prior to autoHCT	
No	71 (54)
Yes	61 (46)

One hundred and eight of the 132 patients (82 %) had received ABVD regimen as a frontline chemotherapy. The vast majority of patients (91 %) received cisplatin-based regimen, DHAP (dexamethasone, cytarabine, cisplatin) or ESHAP (etoposide, methylprednisolone, cytarabine, cisplatin), as first-line salvage chemotherapy. Subsequent lines of salvage treatment included IVE (ifosfamide, etoposide, epirubicin), ICE (ifosfamide, carboplatin, etoposide), dexaBEAM (dexamethasone, carmustine, etoposide, cytarabine, melphalan) or gemcitabine-based regimens. The patients received a median of 1 (range 1–4) salvage chemotherapy line prior to autoHCT. Finally, fifty-nine patients were in CR and sixty-two in PR at autoHCT, respectively. Eleven patients did not respond to the salvage chemotherapy and they underwent autoHCT in less than PR. Pretransplant ^18^FDG-PET was performed in 33 (25 %) of the 132 patients at the time of admission for HDT. Twenty-two of those 33 patients had negative ^18^FDG-PET scans. ^18^FDG-PET was positive in 11 patients.

The autologous graft source was mobilized peripheral blood in 74 % and bone marrow in 18 % of all cases. Eight percent of patients received both bone marrow and mobilized peripheral blood as a source of stem cells. The median number of infused CD34-positive cells was 5.0 × 10^6^ cells/kg (range 2.4–6.7). Engraftment was observed in all but four patients who died within 10 days of autoHCT from infection. Recovery to granulocyte count >0.5 G/l occurred at a median 13 days and platelet count >20 G/l at a median 15 days. Table 2 shows transplant details.

**Table 2:** Transplant details

Characteristics	Number (%)
Disease status at autoHCT	
CR	59 (45)
PR	62 (47)
Less than PR	11 (8)
PET status at autoHCT	
Negative	22 (17)
Positive	11 (8)
Not performed	99 (75)
Autologous graft source	
Mobilized peripheral blood	97 (74)
Bone marrow	24 (18)
Bone marrow and mobilized peripheral blood	11 (8)
Conditioning regimen	
Modified BEAM	132 (100)
Lymphocyte count on day +15 after autoHCT	
Median 380/μl, range 15–2,560/μl	
≤500/μl	92 (70)
>500/μl	31 (23)
Not applicable	4 (3)
Not done	5 (4)

*CR* complete response, *PR* partial response, *autoHCT* autologous haematopoietic stem cell transplantation

### Survival data

**Fig. 1:**
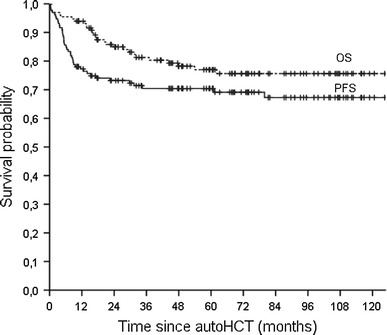
Kaplan–Meier estimates of overall survival (OS) and progression-free survival (PFS) for the whole study group

The median follow-up time of surviving patients is 68 months (range 10–139 months). Figure 1 illustrates the Kaplan–Meier survival curves for the whole study group. At 5 and 10 years after transplantation, estimated OS was 77.0 % (95 % CI 68.3–83.9 %) and 75.6 % (95 % CI 66.8–82.7 %), respectively. The respective PFS rates were 69.1 % (95 % CI 60.3–76.5 %) and 65.6 % (95 % CI 55.9–74.0 %) (Fig. 1).

Patients with refractory HL had similar 5-year OS estimates to those with relapsed disease [77.8 % (95 % CI 69.5–87.4 %) and 71.1 % (95 % CI 55.0–83.2 %), respectively, *p* = 0.46]. The respective 5-year PFS rates were 71.4 % (95 % CI 60.6–80.2 %) and 64.5 % (95 % CI 49.3–77.2 %) (*p* = 0.46).

**Fig. 2:**
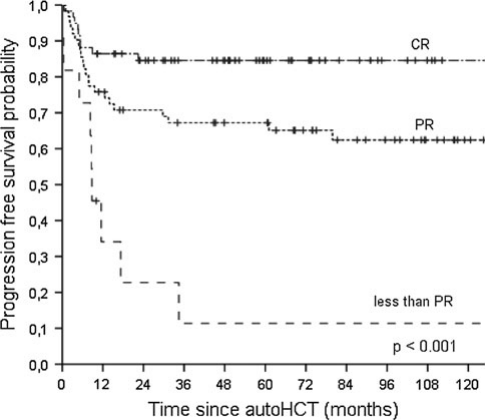
Kaplan–Meier estimates of progression-free survival for patients stratified by the disease status at transplant: complete response (CR), partial response (PR) and less than PR

When patients were stratified by the disease status at transplant, the 5-year OS estimates were 91.0 % (95 % CI 80.7–96.2 %), 71.3 % (95 % CI 58.3–81.6 %) and 27.7 % (95 % CI 8.7–60.7 %) for patients in CR, PR and less than PR, respectively (*p* < 0.001). The respective 5-year PFS rates were 84.6 % (95 % CI 73.3–91.7 %), 65.1 % (95 % CI 52.3–75.9 %) and 11.4 % (95 % CI 2.1–43.5 %) (*p* < 0.001) (Fig. 2).

**Fig. 3:**
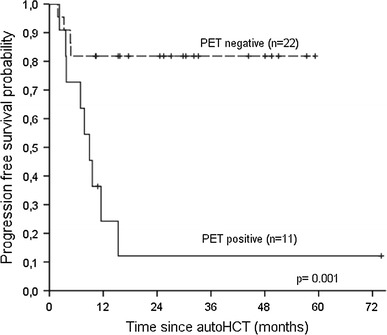
Kaplan–Meier estimates of progression-free survival for patients stratified by pretransplant ^18^FDG positron emission tomography (PET) status

Pretransplant ^18^FDG-PET was available for 33 patients. Two-year OS for patients with negative and positive scans was 90.9 % (95 % CI 72.3–97.5 %) and 77.8 % (95 % CI 45.2–93.7 %), respectively (*p* = 0.22), whereas the respective 2-year PFS was 81.8 % (95 % CI 61.5–92.7 %) and 12.1 % (95 % CI 2.3–45.0 %) (*p* = 0.001). The median PFS was not reached for patients with negative ^18^FDG-PET scans, compared to 9 months for patients with positive status (Fig. 3).

Thirty-four patients experienced relapse or disease progression after autoHCT. The 5-year cumulative incidence of relapse was 26 % (95 % CI 20–35 %). All but one of the 34 relapses occurred within 36 months of autoHCT.

Twenty-eight patients (21 %) in our study have died. The cause of death in 18 patients was relapse/progression of the disease. Four other patients who relapsed after autoHCT died from complications after subsequent allogeneic (*n* = 3) or autologous (*n* = 1) haematopoietic stem cell transplantation. Six patients have died from causes not related to lymphoma relapse/progression. The causes of deaths included infections, veno-occlusive disease (VOD) and second acute myeloid leukemia. The 1-year and 5-year cumulative incidence of NRM was 2 % (95 % CI 1–7 %) and 4 % (95 % CI 1–10 %), respectively. Second malignancy occurred in 3 of the 132 patients, including two acute myeloid leukemias and one acute lymphoblastic leukemia. The second neoplasms developed at a median of 8 years (range 4.7–8.4 years) from autoHCT. The 10-year cumulative incidence of developing a second malignancy was 7 % (95 % CI 2–22 %).

### Prognostic factors analysis

Univariate analysis identified several risk factors for OS and PFS for the whole study group (Table 3). The following factors were found to be significant for OS: age at transplant (<45 vs. ≥45 years) (*p* < 0.001), disease status at transplant (CR vs. less than CR) (*p* = 0.003), number of pretransplant salvage chemotherapy lines (1 vs. >1) (*p* = 0.001) and ALC at 15 ± 1 day after autoHCT (≤500 vs. >500/μl) (*p* = 0.056). Poor PFS was associated with more than one salvage chemotherapy line prior to autoHCT (*p* < 0.001) and disease status at transplant worse than CR (*p* = 0.002). In multivariate analysis, the number of pretransplant salvage chemotherapy lines and disease status at transplant remained significant for both OS and PFS. In addition, OS was significantly impacted by age at transplant (Table 4).

**Table 3:** Univariate analysis of prognostic factors associated with overall survival (OS) and progression-free survival (PFS) for all patients

Group	*N*	5-year OS (95 % CI)	*p*	5-year PFS (95 % CI)	*p*
Clinical stage					
II	29	82.2 (64.6–92.1)	0.79	72.1 (53.8–85.2)	0.83
III–IV	99	75.1 (64.7–83.2)		69.1 (58.9–77.7)	
B symptoms at diagnosis					
No	32	69.4 (48.1–84.7)	0.42	59.2 (40.4–75.6)	0.30
Yes	94	78.1 (67.9–85.7)		72.6 (62.4–80.6)	
Gender					
Male	71	71.5 (58.4–81.7)	0.38	65.3 (52.8–76.0)	0.43
Female	61	79.7 (67.1–88.3)		73.0 (60.5–82.7)	
Age at transplant					
<45 years	111	81.6 (72.6–88.1)	<0.001	73.4 (64.2–80.8)	0.056
≥45 years	21	39.3 (17.4–66.6)		40.2 (18.0–67.4)	
Disease status at transplant					
CR	59	91.0 (80.7–96.2)	0.003	84.6 (73.3–91.7)	0.002
Less than CR	73	72.1 (35.5–76.3)		57.6 (45.6–68.5)	
Number of prior salvage regimens					
1	86	85.1 (75.7–91.3)	0.001	82.1 (72.5–88.9)	<0.001
2 or more	46	51.4 (33.3–69.2)		41.2 (25.1–58.0)	
Radiotherapy before transplant					
No	71	76.1 (63.5–85.4)	0.87	68.3 (56.2–78.4)	0.65
Yes	61	73.7 (60.2–83.9)		68.7 (55.7–79.3)	
Lymphocyte count on day +15					
≤500/μl	92	72.2 (26.2–79.8)	0.056	66.7 (56.0–75.9)	0.335
>500/μl	31	91.3 (47.1–77.0)		79.2 (61.1–90.2)	

*CI* confidence interval, *CR* complete response, *PR* partial response

**Table 4:** Summary of results from overall survival (OS) and progression-free survival (PFS) cox model

Group	OS		PFS	
	HR (95 % CI)	*p*	HR (95 % CI)	*p*
*All patients*				
Number of salvage regimens before HCT				
1 versus 2 or more	2.83 (1.31–6.10)	0.008	3.26 (1.69–6.29)	<0.001
Disease status at transplant				
CR versus less than CR	2.80 (1.04–7.50)	0.030	2.33 (1.09–4.98)	0.029
Age at transplant				
<45 versus ≥45 years	3.52 (1.62–7.67)	0.001		ns
*Patients with relapse*				
Age at transplant				
<45 versus ≥45 years	5.47 (1.65–18.13)	0.005	4.99 (1.48–16.80)	0.010
Remission duration				
<12 versus ≥12 months	3.17 (0.91–19.98)	0.069	3.17 (1.05–9.51)	0.040
Number of salvage regimens before HCT				
1 versus 2 or more		ns	4.40 (1.39–13.90)	0.012
*Patients with refractory disease*				
Number of salvage regimens before HCT				
1 versus 2 or more	3.83 (1.38–10.65)	0.010	3.89 (1.67–9.08)	0.002
Disease status at transplant				
CR versus less than CR	6.43 (0.84–49.50)	0.074	5.24 (1.22–22.48)	0.026

*HR* hazard ratio, *CI* confidence interval, *CR* complete response, *HCT* haematopoietic stem cell transplantation

**Fig. 4:**
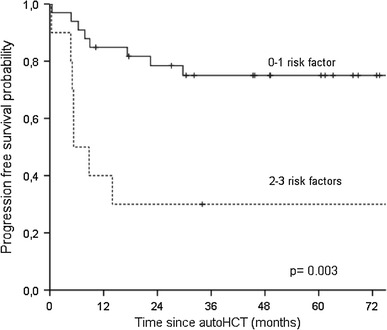
Kaplan–Meier estimates of progression-free survival for patients with relapsed Hodgkin lymphoma stratified by the number of the following risk factors: duration of remission <12 months, age at transplant ≥45 years, and two or more prior salvage therapy lines

Within the group of patients with relapsed disease, univariate analysis revealed that poor OS was associated with age ≥45 years versus <45 years at transplant (5-year OS estimates 37.5 vs. 79.5 %; *p* = 0.003) and ALC ≤500 versus. >500/μl at 15 ± 1 day after autoHCT (5-year OS estimates 62 vs. 100 %; *p* = 0.037). In addition, duration of remission and disease status at transplant tended to impact OS (*p* = 0.082 and 0.080, respectively). PFS was adversely impacted by more than one versus one salvage chemotherapy line prior to transplant (5-year PFS estimates 43 vs. 75 %; *p* = 0.027), the duration of remission <12 versus ≥12 months (5-year PFS estimates 49 vs. 78 %; *p* = 0.025), and age ≥45 years versus <45 years at transplant (5-year PFS estimates 37 vs. 71 %; *p* = 0.073). In multivariate analysis, age at transplant remained significant for both OS and PFS. Additionally, the number of salvage chemotherapy lines and the length of remission were independently prognostic for PFS (Table 4). Having found age ≥45 years, more than one salvage chemotherapy line and duration of remission <12 months as the independent predictors of PFS for patients with relapsed disease, we divided those patients into two groups according to the number of identified independent unfavorable factors for outcome (0–1 vs. 2–3). The median PFS was not reached for patients with 0–1 risk factors (*n* = 64), compared to 5 months for patients with 2–3 risk factors (*n* = 25) (*p* = 0.003) (Fig. 4).

**Fig. 5:**
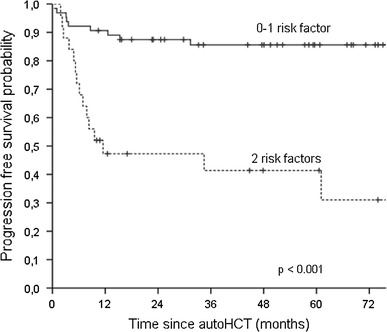
Kaplan–Meier estimates of progression-free survival for patients with refractory Hodgkin lymphoma stratified by the number of the following risk factors: disease status at transplant worse than CR, and two or more prior salvage therapy lines

Among the patients with refractory disease, univariate analysis revealed that worse OS was associated with more than one versus one salvage chemotherapy line prior to autoHCT (5-year OS estimates 45 vs. 89 %; *p* = 0.001), age ≥45 versus <45 years (5-year OS estimates 43 vs. 82 %; *p* = 0.035) and less than CR versus CR at transplant (5-year OS estimates 69 vs. 97 %; *p* = 0.010). Poor PFS was associated with more than one versus one salvage chemotherapy line prior to autoHCT (5-year estimates 37 vs. 86 %; *p* < 0.001) and less than CR versus CR at transplant (61 vs. 93 %; *p* = 0.003). In multivariate analysis, the number of salvage chemotherapy lines and disease status at transplant impacted both OS and PFS (Table 4). Consequently, patients with refractory disease were divided into two groups according to the number of identified independent unfavorable factors for outcome (0–1 vs. 2). The median PFS was not reached for patients with 0–1 risk factor (*n* = 33), compared to 11 months for patients with two risk factors (*n* = 10) (*p* < 0.001) (Fig. 5).

## Discussion

The role of high-dose therapy and autoHCT for patients with relapsed and refractory HL is well defined. In this study of 132 patients with relapsed or refractory HL, we confirmed that the high proportion of these patients can be cured with autoHCT following modified BEAM regimen. Since there are no available published results of prospective trials comparing HDT regimens as a part of autoHCT, the usage of modified BEAM with escalated dose of cytarabine was based on the institutional preference and experience. The long median follow-up time exceeding 5 years has allowed to evaluate the 10-year outcomes of HDT and autoHCT. The 10-year OS and PFS were 76 and 66 % in our study, respectively, which is consistent with reported results of studies published in the last decade, across which the range of PFS was from 60 to 71 % at 5-10 years for patients treated with BEAM-like preparative regimens [12, 16, 20]. Regarding late events, the 10-year cumulative incidence of second malignancies was 7 %, which is in line with the previously published studies. A 5-year CI of second malignancies reported by Sureda was 4.3 % [3] and a 15-year CI reported by Forrest and Goodman was 8 and 15.3 % [21, 22], respectively. Our study confirms that relapse is the main cause of transplant failure, since the 5-year cumulative incidence of relapse exceeded 25 % in the present analysis. It is noteworthy also that more than 95 % of relapses occurred within 3 years of autoHCT.

As previously stated, the long-term outcomes of autoHCT are highly associated with disease sensitivity to pretransplant salvage chemotherapy [10, 11] and the number of salvage regimens [5, 13]. Consistent with other reports, we also observed a major prognostic effect of disease status at HDT and the number of salvage chemotherapy lines on both OS and PFS after transplant. In addition, we identified patient age at transplant as independently affecting OS in our analysis. Age is a well-known prognostic factor at first-line treatment identified by the International Prognostic Factors Project [23], but its impact on outcome after autoHCT has not been clearly defined. Bierman et al. [13] analyzed the impact of prognostic factors included in the International Prognostic Index on the survival of patients with HL treated with autoHCT and confirmed that age, low serum albumin, anemia and lymphocytopenia were independently associated with poorer event-free survival and overall survival after transplant. Sirohi et al. [11] also reported that the International Prognostic Index independently predicts both OS and PFS after autoHCT. In contrast, the other authors reported no association between age and transplant outcomes [5, 12, 24, 25]. Interestingly, we have found age at transplant as independently associated with the outcomes of autoHCT for patients with relapsed HL. In contrast, we did not prove that age affected OS or PFS of patients with refractory disease.

Pretransplant ^18^FDG-PET status was not included in multivariate analysis in the present study, since the group evaluated by PET was small, consisting of 33 patients. However, it is worth pointing out that ^18^FDG-PET was strongly correlated with PFS in univariate analysis. The outcome for ^18^FDG-PET-positive patients was poor with the median PFS of 9 months, which is in agreement with results reported by other authors [14, 15, 18].

In addition to the evaluation of predictive value of different clinical features, we investigated also the impact of early lymphocyte recovery after autoHCT on the outcomes of transplant. Early lymphocyte recovery after autoHCT has been shown to be associated with positive clinical outcome in non-Hodgkin lymphoma [26]. However, there are limited and conflicting data on whether it affects posttransplantation outcome in HL [27–29]. The results of our univariate analysis revealed that ALC >500/mcl at 15 ± 1 day was associated with better OS in the whole study group and in the subgroup of patients with relapsed disease (*p* = 0.056 and 0.037, respectively). In contrast, for patients with refractory disease, no association of this parameter with the outcomes after transplant was found. We concluded that early lymphocyte recovery after autoHCT is associated with better OS of patients with relapsed HL undergoing transplantation, though it does not independently predict better survival after transplant.

Furthermore, we have demonstrated that the number of identified independent adverse prognostic factors is inversely correlated with PFS after autoHCT. For patients with relapsed disease, multivariate analysis revealed that age ≥45 years at transplant, duration of remission <12 months and the number of salvage therapy lines >1 appeared to be independent adverse predictors for PFS. The results of our study indicate that the outcome of autoHCT for patients with 2 or 3 of these factors is very poor with the median PFS below 6 months. Among patients with refractory disease, the outcomes were impacted by the disease status at transplant and the number of salvage therapy lines. Similarly, the outcome of patients with 2 independent risk factors seemed to be not satisfactory with the median PFS of 11 months. Moreover, it is noteworthy that the median PFS was not reached for patients with none or only one risk factor in both refractory and relapsed setting. Our results are in line with the results of several other groups evaluating the correlation between the survival after autoHCT and the number of identified independent risk factors. The results of previously published studies also demonstrated that the presence of two or three different risk factors, such as time to relapse <12 months [4, 9, 30], less than minimal disease at transplant [31], the number of prior chemotherapy regimens [32], extranodal disease [4, 9, 32], B symptoms [4, 30, 31], poor performance status [30] or nodular sclerosis histology [30], is associated with worse outcomes in comparison with the survival of patients with one adverse factor. Our results confirm that the use of distinct clinical features may allow to predict the risk of autoHCT failure. The treatment strategy for patients with two or three adverse clinical prognostic factors remains the area for further studies on new salvage regimens or posttransplant maintenance therapy with novel agents that are non-cross-resistant to chemotherapy. It should be mentioned that no patients in our report received such therapy. The results of studies with agents such as brentuximab vedotin or histone deacetylase inhibitors in this clinical setting are awaited.

In conclusion, the results of the present study support the current standard of HDT followed by autoHCT for patients with relapsed and refractory HL. Despite the limitations of the retrospective study, the use of uniform-modified BEAM regimen and long-term follow-up exceeding 5 years allow a realistic assessment of long-term outcomes and complications after autoHCT. Our study confirms that more than 70 % of patients with relapsed or refractory HL without adverse prognostic factors may be cured with HDT and autoHCT. However, the outcome following autoHCT of patients with two or more risk factors is poor. We believe that the results of our study may be helpful in identification of these higher-risk patients, who may benefit most from the use of novel agents in the pre- or posttransplant setting. The results of our analysis, based on limited number of patients, also suggest that pretransplant ^18^FDG-PET-positive status is associated with extremely poor PFS after autoHCT and support further investigations on optimal treatment options for patients with ^18^FDG-PET-positive status after salvage chemotherapy .
